# Hydrophobia of human rabies

**DOI:** 10.1002/ccr3.1846

**Published:** 2018-10-18

**Authors:** Juliana R. Tongavelona, Rivo Andry Rakotoarivelo, Fy S. Andriamandimby

**Affiliations:** ^1^ Infectious Diseases Universite d'Antananarivo Faculte de Medecine Antananarivo Madagascar; ^2^ Infectious Diseases Universite de Fianarantsoa Fianarantsoa Madagascar; ^3^ Institut Pasteur de Madagascar Virology Antananarivo Madagascar

**Keywords:** human rabies, hydrophobia

## Abstract

Hydrophobia is a clinical sign characteristic of human rabies. This sign occurs following paroxysmal contractions of pharynx responsible for hydrophobic spasms.

A 49‐year‐old man presented to the infectious disease ward, with hydrophobia allowing us to suspect human rabies. He has been bitten to the big toe by a dog 2 months prior to his hospitalization. He would have received a local wound care immediately after exposure. He also received only one dose of rabies postexposure vaccine by ID route 1‐week aftermath.^1^

During the physical examination, the patient was conscious and polypneic at 34 bpm and tachycardia at 105 pulses/min. The body temperature was 37°C, and the blood pressure was 100/70 mm Hg. He was found to have aerophobia. He also complained of intense thirst, but any attempt of water intake caused hydrophobic spasm, described as a blockage in the throat with worsening of dyspnea, and he systematically repelled the glass of water (Video S1). The patient died the day of his admission. Rabies diagnosis was confirmed by direct fluorescent antibody test (DFAT) using postmortem brain samples (Figure 1).

**Figure 1:**
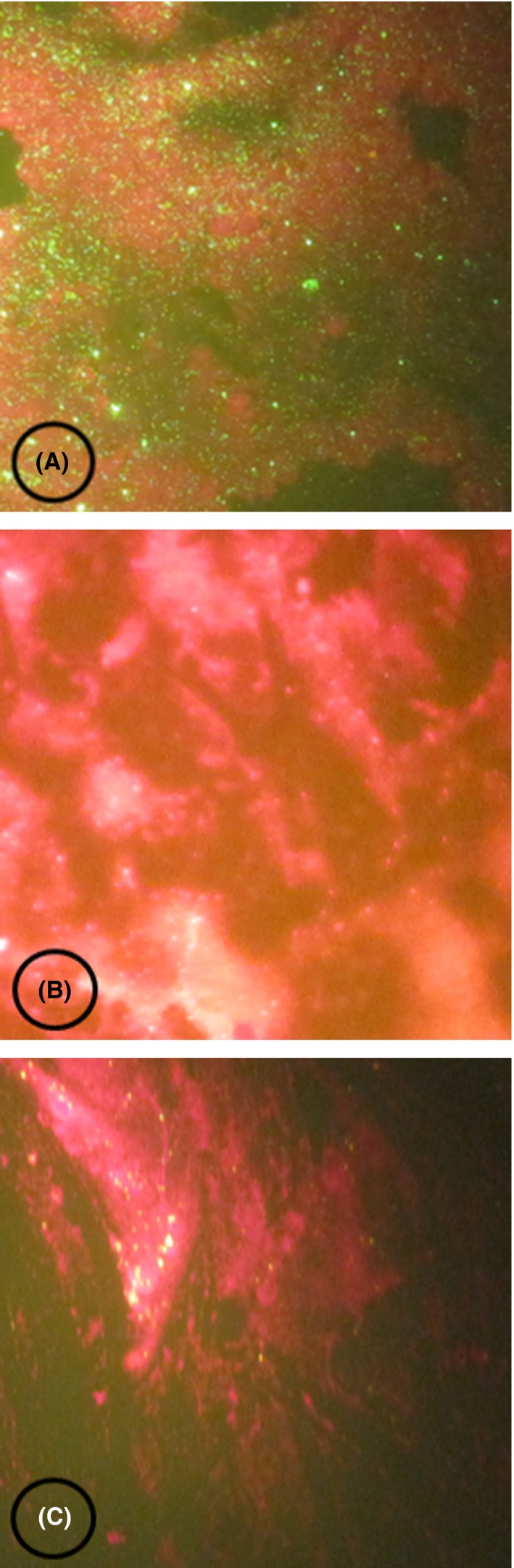
Result of direct fluorescent antibody test detecting rabies nucleoprotein. A, Positive control. B, Negative control. C, Human case

The patient did not receive the four doses of rabies postexposure vaccine recommended by World Health Organization.^1^

Hydrophobia is characteristic of furious rabies. Other classical features of rabies are fluctuating consciousness, modified mental state, aerophobia, phobic or inspiratory spasms, and autonomous stimulation signals. Death occurs usually 5.7 days on patients showing furious rabies after the first symptoms.^2^

## AUTHOR CONTRIBUTIONS

RAR: conceived the original idea. FSA: confirmed the diagnostic by direct fluorescent antibody test. JRT: wrote the manuscript in consultation with RAR and FSA. All authors provided critical feedback and helped shape the manuscript.
